# A kind of universal quantum secret sharing protocol

**DOI:** 10.1038/srep39845

**Published:** 2017-01-12

**Authors:** Xiu-Bo Chen, Zhao Dou, Gang Xu, Xiao-Yu He, Yi-Xian Yang

**Affiliations:** 1 Information Security Center, State Key Laboratory of Networking and Switching Technology, Beijing University of Posts and Telecommunications, Beijing 100876, China; 2 Department of Computer Science, University of Calgary, Calgary, Alberta, T2N 1N4, Canada; 3 School of Software Engineering, Beijing University of Posts and Telecommunications, Beijing 100876, China; 4 School of Computer Science, Beijing University of Posts and Telecommunications, Beijing 100876, China; 5 State Key Laboratory of Public Big Data, Guizhou 550025, China

## Abstract

Universality is an important feature, but less researched in quantum communication protocols. In this paper, a kind of universal quantum secret sharing protocol is investigated. Firstly, we design a quantum secret sharing protocol based on the Borras-Plastino-Batle (BPB) state. Departing from previous research, our protocol has a salient feature in that participants in our protocol only need projective measurement instead of any unitary operations. It makes our protocol more flexible. Secondly, universality of quantum communication protocols is studied for the first time. More specifically, module division of quantum communication protocols and coupling between different modules are discussed. Our aforementioned protocol is analyzed as an example. On one hand, plenty of quantum states (the BPB-class states and the BPB-like-class states, which are proposed in this paper) could be used as carrier to perform our protocol. On the other hand, our protocol also could be regarded as a quantum private comparison protocol with a little revision. These features are rare for quantum communication protocols, and make our protocol more robust. Thirdly, entanglements of the BPB-class states are calculated in the Appendix.

In secret sharing problem, a boss wants to split his secret into several parts, and distribute them to various agents. The secret could be reconstructed by sufficient number of agents. These agents need to cooperate with each other. Classical secret sharing problem is independently introduced by Shamir^1^ and Blakley^2^ in 1979. Quantum secret sharing (QSS) is the quantum version solution of secret sharing problem. Players utilize some necessary quantum technique to achieve the goal. Importantly, quantum mechanics provides the possibility of designing unconditionally secure protocols^3^,^4^.

There are two branches in QSS protocols. The first one is sharing classical information. In 1999, Hillery *et al*.^5^ proposed a QSS scheme with the Greenberger-Horne-Zeilinger (GHZ) state. Later, Guo *et al*. considered a QSS protocol without entanglement^6^. In this protocol, only product states are employed. After that, Xiao *et al*.^7^ generalized Hillery’s scheme^5^ into arbitrary multi-parties, and also increased the efficiency of their scheme. In 2005, a QSS between multiparty (*m* members in group 1) and multiparty (*n* members in group 2) without entanglement was investigated by Yan *et al*.^8^. Only single photons are used in the protocol. The secret message shared by all members of group 1 is shared by all members of group 2. Only the entire set of each group (not only group 1 but also group 2) is efficient to read the secret message. Afterwards, a dynamic QSS protocol was considered^9^. The secret is shared between a sender and a dynamic agent group. Dynamic schemes are more flexible and suitable for practical applications. In the same year, Long *et al*.^10^ designed a QSS protocol via the BPB state. In the protocol, the boss owns three particles in one state, while each of three agents only owns one. Unfortunately, Qin *et al*.^11^ found that the information leakage exists in above protocol. Then, Dehkordi *et al*.^12^ designed a (*t, m*) − (*s, n*) threshold QSS scheme between multiparty (*m* members in group 1) and multiparty (*n* members in group 2) using GHZ state. Threshold scheme is useful and efficient when parties are not all present. Recently, a QSS protocol based on local distinguishability is proposed^13^. The protocol also is a (*k, n*) threshold scheme (*k* = 2 or 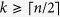).

The other branch is sharing quantum information, i.e., quantum state. This kind of protocol is also be called as quantum state sharing (QSTS). Cleve *et al*.^14^ firstly introduced the concept of QSTS in 1999. Later, a multi-party *m*-particles separable state sharing protocol was studied by Yang *et al*.^15^. A short time ago, a sequential multi-qubit secret sharing protocol was given by Ray *et al*.^16^. The sequential secret sharing means that the dealer can add some secret states in the middle of processes, without performing the protocol again.

Lately, Zhang *et al*. designed a quantum summation protocol^17^ and quantum private comparison (QPC) protocol^18^ based on the BPB state, respectively. If each of three participants measures two particles respectively in one BPB state, three results are relevant. This correlation is the key of these two protocols. It also ensures the correctness and security of the protocols.

The universality is one of the crucial characters in practical situation. In computer science, universality is researched and shown in the form of module division^19^,^20^. Concretely, a system is divided into different modules, coupling is the degree of interdependence between different modules. In a well-designed system, one module should not be strongly connected with the others. When the other modules are determined, this module is better to be alterable. Lowering coupling will reduce the connection of different modules, and the impact of revising one module to the whole system. It makes the system more robust.

For a quantum communication protocol, state is regarded as a part of modules. If a protocol can be performed by different states, it’s universal. In 2014, we^21^ considered the universality of a QPC protocol originally. Concretely, we discussed how to perform one protocol in different quantum states. We researched the symmetry of some quantum states, and proposed a class of QPC protocols based on it^21^. This is the first universal QPC protocol class, and it is easy to be performed in the existing technical condition. The protocol which belongs to this class can be performed by lots of symmetrical states with a little revision.

Entanglement is one of the most important property of quantum mechanics and quantum information processing. Entanglement channel is an important tool for quantum information processing^5^,^7^,^13^,^21^. Highly entangled states are vital for the establishment of entanglement channel. For bipartite quantum state, entanglement can be easily calculated by reduced density matrix and von Neumann entropy. However, there is no recognized method to calculate the entanglement of a multipartite state. In 2005, Eisert *et al*.^22^ summarized some candidate methods of multi-particle entanglement. One of the candidates is proposed by Wei *et al*.^23^. They utilized the geometric measure to describe the multi-particle entanglement.

In this paper, we firstly follow the works in refs 17, 18, and design a QSS protocol based on the BPB state. In our protocol, the correlation of the measurement result is also exploited. What’s more, the boss only need to prepare the quantum state, transport the state to Alice and Bob, and perform measurement. While, agents only need to perform measurement. The procedures are simple, and easy to be achieved under current conditions.

Secondly, we research the universality of quantum communication protocols in detail. First of all, module division of quantum communication protocol is proposed. There are seven modules in total. Coupling of different modules are discussed. Some existing protocols are analyzed as example. We say that if coupling of different modules in a protocol is low, the protocol is universal. Subsequently, peculiarity of the BPB state is studied, and the BPB-class/BPB-like-class states are proposed correspondingly. In these two classes, all of states have the similar form, which are detailed in equation (7) and (10). We extend aforementioned protocol into a class of QSS protocols based on the BPB-class/BPB-like-class states. This is the first universal QSS protocol class. The protocol can be performed by lots of states. All the states of the above two classes, can be utilized to perform the protocol perfectly. Next, we discuss the relation of the QPC protocol^18^ to our QSS protocol. We find that the private comparison is the inverse process of secret sharing in a way. If we have designed a QPC protocol, it may be easy to infer a QSS protocol. It shows that our protocol can be utilized to accomplish the task of QPC or QSS with a little alteration. Through these analysis, we find that our protocol has low coupling, and is robust.

Thirdly, entanglements of the six-particle BPB-class states are investigated by *pseudo entanglement* and Wei’s tool^23^ respectively. Pseudo entanglement is introduced by ourselves and inspired by the way to calculate the entanglement of bipartite quantum state. Detailed calculation is given in Supplementary Information.

## Results

### A new quantum secret sharing protocol based on |Ψ_6*qb*_〉

#### Preliminaries

In 2007, Borras *et al*.^24^ discovered a new six-particle state, which is maximum entangled (it’s called BPB state for short). The state is known as


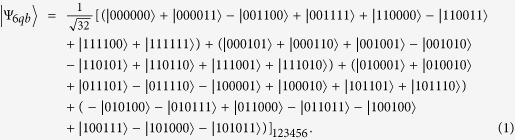


This state can be rewritten in *X* basis as:


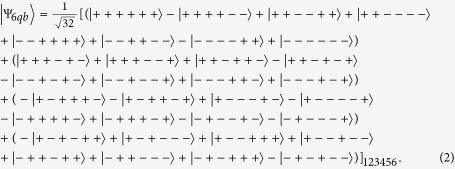


For further research, the state could also be rewritten as


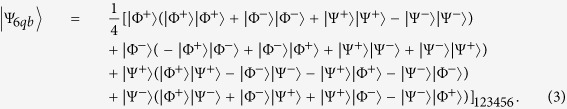


Here,


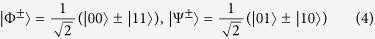


are Bell states. These four states can be used to construct an orthonormal basis, i.e., Bell basis.

We can encode four Bell states into two classical bits:


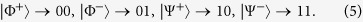


Furthermore, according to Eq. (3), if we measure the (*1*-*st, 2*-*nd*), (*3*-*rd, 4*-*th*), (*5*-*th, 6*-*th*) particle of one BPB state by Bell basis, and encode the result into two classical bits accordingly, we can find that 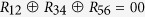. For instance, if the results are |Φ^−^〉, |Ψ^+^〉 and |Ψ^−^〉, then *R*_12_ = 01, *R*_34_ = 10 and *R*_56_ = 11. This equation is inspired by and similar with Zhang’s protocol^17^,^18^. The differences are orders of the *5*-*th* and *6*-*th* particle.

In other words, exact measurement results of the BPB state cannot be predetermined, but the relation of three results is satisfied anyway. It shows an excellent property, which can be utilized to design some quantum communication protocols.

Suppose that the Boss Charlie owns the secret messages *S*. The binary representation of *S* is *S* = (*s*_0_, *s*_1_, …, *s*_2*N*−1_), i.e, *S* could be rewritten as 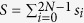. For further consideration, we split *S* into pairs, namely, *S* = (*S*_0_, *S*_1_, …, *S*_*N*−1_) = ((*s*_0_, *s*_1_), (*s*_2_, *s*_3_), …, (*s*_2*N*−2_, *s*_2*N*−1_)).

#### The processes of our quantum secret sharing protocol

[*S* − 1] The boss Charlie prepares the *N* groups of |Ψ_6*qb*_〉 state. He divides the groups into three sequences. The first and second particles of each state form the sequence 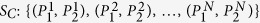. Similarly, the third and fourth (fifth and sixth) particles form the sequence 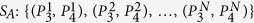 (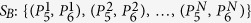).

[*S* − 2] Then, Charlie produces two decoy state sequences. The state in the sequences belongs to the set {|0〉, |1〉, |+〉, |−〉}, and the length of each sequence is *K*. As soon as the sequences are produced, Charlie inserts the first (second) decoy state sequence into *S*_*A*_ (*S*_*B*_) at the random position. New sequence is symbolized by *S*_*A*2_ (*S*_*B*2_). After that, he sends these two new sequences to agent Alice and agent Bob respectively.

[*S* − 3] Once Alice and Bob received the sequences, three participants check whether there exists an eavesdropper in the channel. Charlie tells them the exact position of decoy state in sequences *S*_*A*2_ and *S*_*B*2_, and what basis they need to utilize in the measurement operation. Actually, they measure the state in *Z* basis if the state is |0〉 or |1〉, and measure the state in *X* basis if it is |+〉 or |−〉. After the measurement, two agents tell Charlie the results, which are necessary for him to analyze the error rate. If the rate is higher than the preset threshold, three participants discard all the sequences and restart the step [*S* − 1]. Otherwise, they go next.

[*S* − 4] Three participants throw these decoy states away, and construct the rest of the particles into sequences *S*_*A*3_, *S*_*B*3_ and *S*_*C*3_ respectively. Afterwards, they measure the (*1*-*st, 2*-*nd*), (*3*-*rd, 4*-*th*), (*5*-*th, 6*-*th*) particles of these sequences in Bell basis, and record the results in two classical bits as the way in Eq. (5). The *i*-th classical bit pairs of Alice, Bob, and Charlie are denoted as *RA*_*i*_, *RB*_*i*_, *RC*_*i*_. That is to say, *RA*_*i*_, *RB*_*i*_, *RC*_*i*_ ∈ {00, 01, 10, 11} for ∀*i*.

[*S* − 5] Then, Charlie calculates *CS*_*i*_ = *RC*_*i*_ ⊕ *S*_*i*_, and announces the sequence *CS*. After the reception of *CS*, Alice and Bob consult and determine one party to rebuild sequence *S*. Suppose that Alice rebuilds the secret, Bob will send *RB* to her. Then, Alice computes 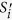 = *CS*_*i*_ ⊕ *RA*_*i*_ ⊕ *RB*_*i*_ = *S*_*i*_. Finally, she can get the secret *S*.

**Figure 1:**
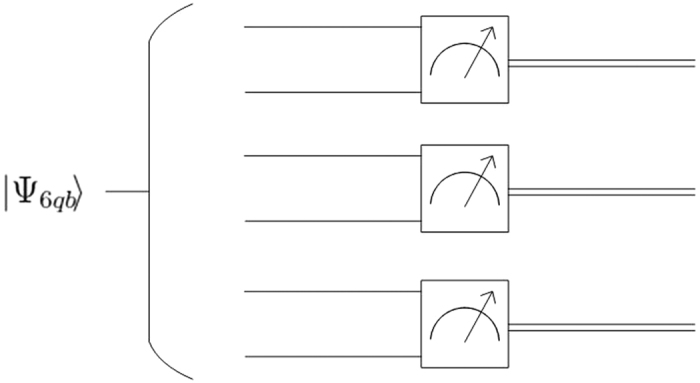
The circuit of a new QSS protocol based on |Ψ_6*qb*_〉.

The processes of our protocol are also graphically described in Fig. 1.

Now, we analyze the correctness of this protocol briefly. As we all know, *RA*_*i*_ ⊕ *RB*_*i*_ ⊕ *RC*_*i*_ = 00. So it’s easy to verify that 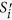 = *CS*_*i*_ ⊕ *RA*_*i*_ ⊕ *RB*_*i*_ = *RC*_*i*_ ⊕ *S*_*i*_ ⊕ *RA*_*i*_ ⊕ *RB*_*i*_ = *S*_*i*_. Namely, 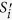 = *S*_*i*_, then we know that Alice has gained the secret successfully. In the Table 1, a part of possible values of classical bits used in our protocol are listed. We also can get 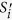 = *S*_*i*_ from this table.

#### Security Analysis

On one hand, outside attack is analyzed. In our protocol, quantum states are only transmitted in [*S* − 2]. In this step, as far as the general outside attack is concerned, the sender use the decoy states to prevent from eavesdropping. Several attacks, such as intercept-resend attack, measurement-resend attack, and entanglement-measure attack, will be detected with a nonzero probability. This conclusion has been proved^25^. Consider Eve’s correlation-elicitation attack, the utilization of decoy states also can ensure the security. This fact has been showed^26^.

Some other common attacks could be resisted with the help of corresponding equipment. Consider about the faked states attack^27^,^28^,^29^,^30^ and time-shift attack^29^,^31^, an extra detector could be utilized to monitor the time when the state arrives at the sides of receiver Alice and Bob. As far as the detector blinding attack^28^,^32^, light intensity monitor will play a vital role.

What’s more, the states are only transported for one time. It shows that the Trojan-horse attacks (such as the delay-photon Trojan horse attack and the invisible photon eavesdropping (IPE) Trojan horse attack) are invalid for our protocol.

To wit: our protocol is safe for the kinds of outside attack.

On the other hand, Participants’ attack is discussed. Consider that all the participants are rational. They will not choose the attack type which cannot help them to gain useful information. For example, they will not disrupt the procession of the protocol deliberately if they could obtain no useful information about the sharing secret.

Since Alice and Bob play the same role in the protocol, we only discuss the situation that Alice wants to get the secret without the help of Bob.

The analysis of reduced density matrix is a common attack approach. Players can use the particles in hand to deduce the residual particles in other participants’ hand, and the classical information of them. In fact, players don’t need to perform any operation but projection measurement. In detail, they only perform measurement on the step [*S* − 4] in Bell basis beside the eavesdropping check. The states before three participants’ final measurement can be only in |Ψ_6*qb*_〉.

The reduced density matrix of Alice’s particles is


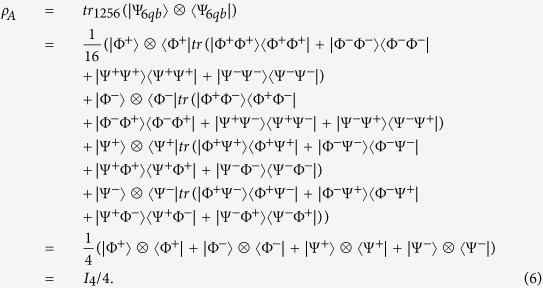


Here, *I*_4_ is the identity matrix of 4 dimension Hilbert space. We can know that *ρ*_*A*_ is independent with players’ classical information *RA*_*i*_, *RB*_*i*_ and *RC*_*i*_.

Similarly, we can find that *ρ*_*B*_ = *ρ*_*C*_ = *I*_4_/4. And then, we know that Alice cannot distinguish Bob’s classical bit 0 from 1, or infer any useful information. So do Bob.

### Universality of quantum communication protocols and our QSS protocol

The universality of quantum communication protocols and our QSS protocol is researched in this section. Firstly, the module division of quantum communication protocols, coupling of different modules, and relative explanations are studied originally. Then, we propose the BPB-class/BPB-like-class states, and extend our QSS protocol into a class of QSS protocols. It shows that our protocol is universal for the module *Quantum States*. Next, we study the relation of our QSS protocol to the QPC protocol^18^. It means that our protocol is universal for the module *Aim*. Finally, discussion about the universality, and a quantitative way to describe the universality are also given. These discussions make our analysis more systematic and comprehensive.

#### The module division of quantum communication protocols

Just like a machine, or software, a quantum communication protocol also can be treated as a system, and be divided into several modules. These modules can uniquely determine a protocol. If all the modules are determined, the protocol is only. A quantum communication protocol consists of:

1. *Aim*: What the communication protocol is designed for.
2. *Participants*: The parties /roles who perform the protocol.
3. *Quantum States*: The carrier of information, which will be transported in the protocol.
4. *Quantum Operations*: The ordered set of quantum operations used in the protocol, by which information is mainly transmitted.
5. *Quantum Equipment*: The equipment for quantum information processing.
6. *Classical Operations* (if necessary): The ordered set of classical operations used in the protocol, by which classical information is transmitted.
7. *Classical Equipment* (if necessary): The equipment for classical information processing.

**Figure 2:**
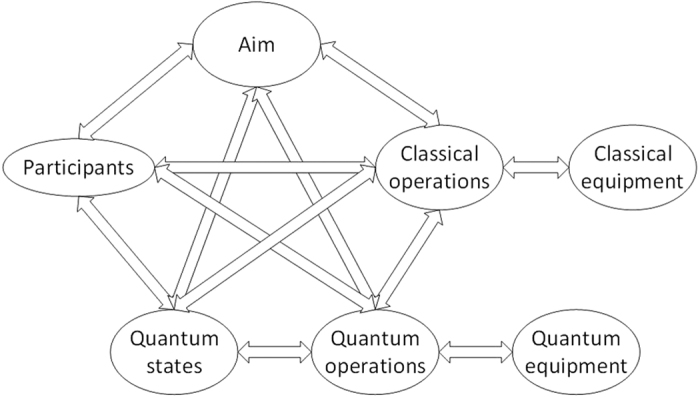
Modules of a quantum communication protocol.

The division of our protocol is also given in Fig. 2. Note that quantum equipment is only related to quantum operations, so the arrow associate with *Quantum Equipment* only exists in *Quantum Operations*. So do the arrow between *Classical Equipment* and *Classical Operations*.

Coupling is the degree to evaluate the relationship between different modules. If the relation is close, we say these modules are highly coupling. In this subsubsection, we consider the coupling of different modules in quantum communication protocols. Before that, we divide the degree of coupling into the following 9 levels, which are shown in Table 2.

If the level of *Participants* → *Aim* is −4, from Table 2, we can know that *Participants* is controlled by *Aim*. Further, we say *Aim* is controlling *Participants*, and the level of *Aim* → *Participants* is +4. A table is given to describe the coupling between different modules in general protocols, i.e., Table 3.

(1) The relationship between *Aim* and the others.

*Aim* → *Participants*, +4. Aim is the core of a protocol. The other modules are designed to achieve the aim. Once the aim is made certain, participants are generally determined. We say that *Aim* and *Participants* are highly coupled. In order to show this point, a brief example is given: The millionaire Alice and Bob are necessary in a QPC protocol. Besides, TP is designed to help the players in most of these protocols. There are three kinds of TP: honest, semi-honest^21^,^26^,^33^,^34^ and dishonest^35^. Different protocols may contain different kinds of TP (Researchers usually don’t employ the honest TP in a protocol because this assumption is too strong).

*Aim* → *Quantum States*, +2. On one hand, lots of quantum states could be utilized to accomplish the same aim. For instance, the GHZ state^26^,^33^, the |*χ*〉 state^21^, and lots of other quantum state^34^,^35^, could be used to perform a QPC protocol. On the other hand, the GHZ state could be exploited to execute not only a QPC protocol, but also a QSS protocol^7^, and so on ref. 36.

*Aim* → *Quantum Operations*, +3. For a certain aim, quantum operations are closely determined. Take remote state preparing (RSP) protocols as example, sharing of some entangled states, measurement of quantum states are necessary^37^,^38^.

*Aim* → *Quantum Equipment*, 0. Quantum equipment is only relative with corresponding quantum operations, so the relation level is 0.

*Aim* → *Classical Operations*, +3. Just like quantum operations, classical operations are also closely determined by the aim. For a RSP protocol^37^,^38^, coding and transmission of measurement results are necessary (Receiver need to use these classical bits to prepare the state).

*Aim* → *Classical Equipment*, 0. Since we say classical equipment is only relative with corresponding classical operations, the relation level is 0.

(2) The relationship between *Participants* and the others.

*Participants* → *Quantum States*. Since *Participants* and *Aim* are highly coupled, the level of *Participants* → *Quantum States* equals to *Aim* → *Quantum States*. Similarly, *Participants* → *QO/QE/CO/CE* equals to *Aim* → *QO/QE/CO/CE*.

(3) The relationship between *Quantum States* and the others.

*Quantum States* → *Quantum Operations*, +1. For the same quantum state, plenty of quantum operations could be performed. In |*χ*〉 state’s case^21^, measurement in *X* basis, *Z* basis, Bell basis, and the others are all allowed. At the same time, Pauli operations are universal for all the bipartite quantum states.

*Quantum States* → *Quantum Equipment*, 0. Similar to the reason we have explained in *Aim* → *Quantum Equipment*, the level of *Quantum States* → *Quantum Equipment* is 0.

*Quantum States* → *Classical Operations*, +1. These two modules have weak coupling, because we cannot derive effective information about classical operations from quantum states, and vice versa.

*Quantum States* → *Classical Equipment*, 0. Likewise, the level of *Quantum States* → *Classical Equipment* is 0.

(4) The relationship between *Quantum Operations* and the others.

*Quantum Operations* → *Quantum Equipment*, +2. Once the quantum operations are made certain, the sort of quantum equipment is determined, but details about the equipment are not. Under many circumstances^39^, security proofs assume that participants have well control of the state preparation and of the measurement devices. But its not necessary for all the protocols. In 2012, Lo *et al*.^40^ proposed a measurement-device-independent (MDI) quantum key distribution (QKD) protocol. In this protocol, measurement device is independent, participants don’t need to know detailed knowledge of measurement devices, or trust them. It’s more safe than the previous protocols because the security is not based on measurement device. MDI protocol can be treated as the protocol in which *Quantum Equipment* does not have strong connection with *Quantum Operations*.

*Quantum Operations* → *Classical Operations*, +2. Sometimes the accomplishment of quantum operations needs the assist of classical information. For instance, in QSTS protocols, agent who recover the state needs the classical information of other agents. So others will convey classical information to him/her. The degree of coupling between *Quantum Operations* and *Classical Operations* is not low.

*Quantum Operations* → *Classical Equipment*, 0. These two modules have no connection, the level is 0.

(5) The relationship between *Quantum Equipment* and the others. As we have described, all the levels are equal to 0 except *Quantum Operations* → *Quantum Equipment*.

(6) The relationship between *Classical Operations* and the others.

*Classical Operations* → *Classical Equipment*, +2. There are two kinds of classical operations in a quantum communication protocol: transmission and storing. On one hand, when we discuss the transmission of classical information, in most cases, we suppose this process is public. The security of this process is not required. On the other hand, storing of classical information is enough safe under the present conditions if we don’t send the information. Based on the analysis above, *Classical Equipment* has a weak connection with *Classical Operations*.

#### A class of QSS protocols via the BPB-class/BPB-like-class states

Inspired by the BPB state, we build a set to describe some states which share common traits. The state in this set is described as follows:


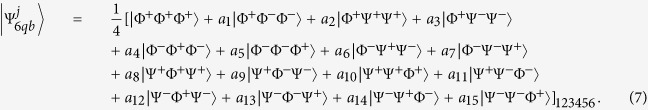


Here, *a*_*i*_ ∈ {1, −1} for 0 ≤ *i* ≤ 15. Consider the global phrase, we suppose that *a*_0_ = 1. There are 2^15^ possible states in the set, all of them could be utilized to perform this protocol, i.e., 1 ≤ *j* ≤ 2^15^. In the Table 4, some states of 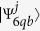 and corresponding coefficients are shown. Here, 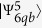 is the quantum carrier in our original QSS protocol.

If we utilize Bell basis to measure 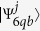, and encode the results of (*1*-*st, 2*-*nd*), (*3*-*rd, 4*-*th*), (*5*-*th, 6*-*th*) particle as *mc*_112_, *mc*_134_, *mc*_156_, respectively, it’s easy to get


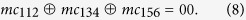


Then, we concatenate them as *mc*_1_ (*mc*_1_ = *mc*_112_||*mc*_134_||*mc*_156_). All the different *mc*_1_ constitute a set *MC*, and


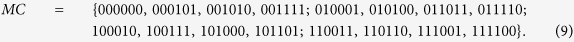


Similarly, there are 2^15^ other states which can also perform this protocol. These states are written in *Z* basis as follow:


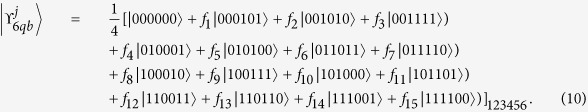


For 0 ≤ *i* ≤ 15, *f*_*i*_ ∈ {1, −1}. Consider the global phrase, we can set *f*_0_ = 1; i.e., 1 ≤ *j* ≤ 2^15^.

Besides, we also can re-encode four states {|00〉, |01〉, |10〉, |11〉} into two classical bits:


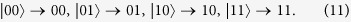


If we utilize *Z* basis to measure 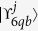, and encode the results as the previous way, these classical bits are denoted as *mc*_212_, *mc*_234_, *mc*_256_, separately. Similarly, we can get


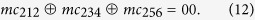


We concatenate these bits as *mc*_2_. Obviously, *mc*_2_ ∈ *MC*, too.

Actually, we call 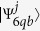 states as the BPB-class states, 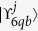 states as the BPB-like-class states.

All the BPB-class states and BPB-like-class states can be utilized to perform our QSS protocol. Our protocol is universal for quantum states. We call these protocols (with different states) as *a class of QSS protocols*. The processes of these protocols are the same with the steps [*S* − 1] to [*S* − 5]. The only difference is, if the BPB-like-class states are utilized, participants need to measure *SA*_3_/*SB*_3_/*SC*_3_ in *Z* basis instead of Bell basis. By the way, the measurement in *Z* basis is much easier to be performed than in Bell basis.

#### The relation of Zhang’s QPC protocol to our QSS protocol

With the development of computer science and corresponding applications^41^,^42^,^43^,^44^,^45^,^46^, security of data has been attracting a lot of attention. Secure multi-party computation (SMC) is an important kind of multi-user remote coordinative computation problems. A good solution of SMC problems need to ensure the correctness of computation and the security of players’ data. In other words, the schemes not only need to help players to get the correct result, but also ensure that the private input values will not be revealed to others.

Private comparison is an important SMC problem. In this problem, two or more players want to compare the equality of the private information. QPC protocol is the quantum version solution of private comparison problem. Zhang *et al*. proposed a QPC protocol based on the BPB state^18^, which could be used to compare three values *M*_1_, *M*_2_ and *M*_3_ for one time. In this subsubsection, we discuss the relation between this QPC protocol and our QSS protocol.

From Table 5, we know that the QPC protocol and QSS protocol we discussed are similar. For ease of analysis, we show the correspondence of these symbols firstly in Table 6.

Analysis of Table 5 is shown as follow. There is almost a one-to-one correlation between the quantum steps (1)–(4), (7) in the QPC protocol and the steps (1)–(5) in our QSS protocol. So do the classical steps (1)–(3) in the QPC protocol and the steps (1)–(3) in our QSS protocol.

On one hand, since the QPC protocol^18^ is designed for three parties without any fourth party, *P*_1_ is not a common player, but a special one who has mission to prepare the states. He could be regarded as the combination of a common player who want to compare his private input, and an assistant player who need to help common player to compare their inputs. Because of that, he also has the motivation to steal more information for his own benefit. That is what the quantum steps (5)–(6), and classical steps (4)–(6) aim for. Concretely, these steps are performed to check whether *P*_1_ shares genuine |Ψ_6*qb*_〉 to *P*_2_ and *P*_3_. Since the Boss Charlie does not have motivation to cheat in QSS protocol, these steps are unnecessary. In this sense, quantum operations of these two protocols are equivalent.

On the other hand, secret sharing could be regarded as the process of diving the information (Boss divides the secret into two/many parts), while private comparison could be considered as the process of integrating information (Assistant player integrates two secret inputs into one bit, i.e., these inputs are equal or not). We can say that the private comparison is the inverse process of secret sharing in a way.

#### A brief discussion about the quantum carrier

All the Bell (*Z*) basis measurement results of the BPB-class (BPB-like-class) states have the same statistical distribution. Firstly, the Bell (*Z*) measurement results of each BPB-class (BPB-like-class) state are related, and shown in Eqs (8) and (12). Secondly, the relations of results are the same for different states, and could be utilized for different functions, such as QSS and QPC. These properties are the key to design a protocol, which is universal for the quantum carrier and aim. Besides that, our protocol is the first of its kind. Thirdly, BPB state has be researched on designing QSS^10^, quantum summation^17^ and QPC^18^ protocol respectively. This six-particle state is a small hot topic on the research. Since all the BPB-class/BPB-like-class states hold the similar properties, these states could also play important roles in quantum communication protocols. In this view, these states are worthy and advantageous for the concern of universality.

#### Discussion about the universality

The situation what we are mostly focus on in this paper is, in general quantum communication protocols, if module *Participants, QO, QE, CO* and *CE* are certain, the module *Aim* and *QS* are determined generally. Namely, it’s hard for researchers to seek a new aim or quantum state to replace the old one in a certain protocol.

In our protocol, quantum states are optional in the foregoing state classes. Participants will have many options to consider. Connection between the third module and the others is weak. This property is discussed in Section *A class of QSS protocols via the BPB*-*class*/*BPB*-*like*-*class states*. Besides, the aim could be QSS or QPC. The difference between the QSS protocol (in this paper) and QPC protocol^18^ is a little. A QSS protocol could be used to execute the QPC protocol with a little modification. Connection between the first module and the others is not particularly strong as other quantum communication protocols. This property is discussed in Section *The relation of the QPC protocol*^18^ *to our QSS protocol*. This is a feature what most of protocols don’t have. This property makes our system more useful. Exactly, our protocol is multi-functional.

In one word, our protocol is subtly designed, and has low coupling.

Besides that, in Table 3 we described the coupling of quantum communication protocols in general. Actually, we can tabulate for any specific protocol. Our QSS protocol is also analyzed as example in Table 7.

A way to quantitatively describe the coupling of a protocol, is summing the absolute value of all the numbers, and then dividing the sum of absolute value in Table 3.


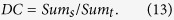


Here, *Sum*_*s*_ means the summation of all the absolute value in a specific protocol, *Sum*_*t*_ means the summation of all the absolute value in Table 3.

Since *Sum*_*t*_ = 56, Eq. (13) could be rewritten as:


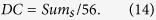


Now, let’s sum the absolute value of all the numbers in Table 7, the answer is 50. So, *DC* = 50/56 = 0.89. It shows that our protocol is well-designed indeed.

In addition, *Sum*_*s*_ and *Sum*_*t*_ also could be the weighted sum of absolute values, not only direct sum. The item which is more valued for the researchers, will have a heavier weight.

Further, for any specific protocol, if *DC* is lower, the coupling of the protocol will be lower, and the protocol will be better-designed. By the way, for all the protocols, *DC* is in direct proportion to *Sum*_*t*_. If we want to compare the coupling of two protocols, the concrete value of *Sum*_*t*_ is not important.

## Discussion

In this paper, we propose a kind of universal QSS protocol. Firstly, we perfectly design a new QSS protocol via the BPB state. Correctness and security analysis of the protocol are given. Only preparation and measurement of the states are needed in the protocol. It shows that the protocol is not only safe, but also flexible. Then, through research on the characteristics of BPB state, the BPB-class states are proposed. Measure of entanglement of these states is also detailedly calculated. Furthermore, the above-mentioned protocol is extended into a class of QSS protocols based on the BPB-class states. In this class, utilized states are alterable. Besides that, we discuss the relation of the QPC protocol^18^ to our protocol, and pioneer the module division and coupling of quantum communication protocols. Some existing protocols are analyzed as example. A way to quantitatively describe the coupling is given. It concludes that our protocol is universal. Our research will help us to find and design universal protocols, which will be more practical under the present technical conditions.

## Methods

### Geometric measure

For a pure state |*ψ*〉, we need to find a separable state |*ϕ*〉 which makes the inner product 〈*ϕ*|*ψ*〉 maximum. The measure of entanglement 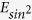 is defined as follows^23^:


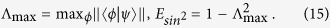


If 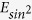 is smaller, 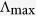 will be larger. Then, |*ψ*〉 is more *like* the pure state |*ϕ*〉, entanglement of |*ψ*〉 is lower. On the contrary, if 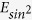 is more close to 1, entanglement of |*ψ*〉 will be higher.

## Additional Information

**How to cite this article**: Chen, X.-B. *et al*. A kind of universal quantum secret sharing protocol. *Sci. Rep.* **7**, 39845; doi: 10.1038/srep39845 (2017).

**Publisher's note:** Springer Nature remains neutral with regard to jurisdictional claims in published maps and institutional affiliations.

## Supplementary Material

Supplementary Information

## Figures and Tables

**Table 1:** A part of possible values of classical bits.

*RA*_*i*_	*RB*_*i*_	*RC*_*i*_	*S*_*i*_	*CS*_*i*_	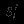	*RA*_*i*_	*RB*_*i*_	*RC*_*i*_	*S*_*i*_	*CS*_*i*_	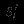
01	11	10	00	10	00	10	10	00	00	00	00
01	11	10	01	11	01	10	10	00	01	01	01
01	11	10	10	00	10	10	10	00	10	10	10
01	11	10	11	01	11	10	10	00	11	11	11
01	10	11	00	11	00	10	11	01	00	01	00
01	10	11	01	10	01	10	11	01	01	00	01
01	10	11	10	01	10	10	11	01	10	11	10
01	10	11	11	00	11	10	11	01	11	10	11

Suppose that *S*_*i*_ = 01, at the same time, *RA*_*i*_ = 10, *RB*_*i*_ = 00, *RC*_*i*_ = 10. We can infer that *CS*_*i*_ = *RC*_*i*_ ⊕ *S*_*i*_ = 10 ⊕ 01 = 11, 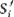 = *RA*_*i*_ ⊕ *RB*_*i*_ ⊕ *CS*_*i*_ = 10 ⊕ 00 ⊕ 11 = 01. So, 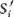 = *S*_*i*_. The correctness is verified.

**Table 2:** The levels of coupling.

0	−1	−2	−3	−4
Irrelevant	A Little Controlled	Partly Controlled	Closely Controlled	Controlled
	+1	+2	+3	+4
	A Little Controlling	Partly Controlling	Closely Controlling	Controlling

**Table 3:** The coupling between different modules.

	*Aim*	*P*	*QS*	*QO*	*QE*	*CO*	*CE*
*Aim*	N/A	+4	+2	+3	0	+3	0
*P*	−4	N/A	+2	+3	0	+3	0
*QS*	−2	−2	N/A	+1	0	+1	0
*QO*	−3	−3	−1	N/A	+2	+2	0
*QE*	0	0	0	−2	N/A	0	0
*CO*	−3	−3	−1	−2	0	N/A	+2
*CE*	0	0	0	0	0	−2	N/A

Note that *P* = *Participants, QS* = *Quantum States, QO* = *Quantum Operations, QE* = *Quantum Equipment, CO* = *Classical operations* and *CE* = *Classical Equipment*. We explain situations *J* → *K* and *K* → *J* only once (Here, *J* and *K* represent different modules). Detailed explanations about this table are given. Since there exist countless protocols up to now, explanations are given in the form of examples.

**Table 4:** Some states of the 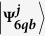 .

The state	*a*_1_	*a*_2_	*a*_3_	*a*_4_	*a*_5_	*a*_6_	*a*_7_	*a*_8_	*a*_9_	*a*_10_	*a*_11_	*a*_12_	*a*_13_	*a*_14_	*a*_15_
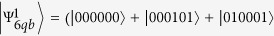 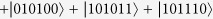 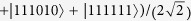	1	1	1	1	1	1	1	1	1	1	1	1	1	1	1
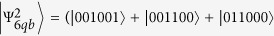 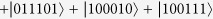 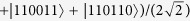	−1	1	−1	1	−1	1	−1	1	−1	1	−1	1	−1	1	−1
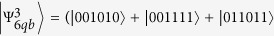 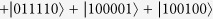 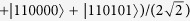	1	1	1	−1	−1	−1	−1	1	1	1	1	−1	−1	−1	−1
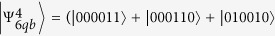 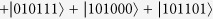 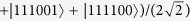	−1	1	−1	−1	1	−1	1	1	−1	1	−1	−1	1	−1	1
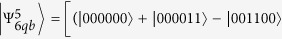 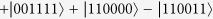 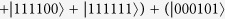 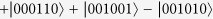 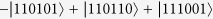 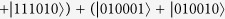 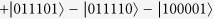 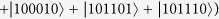 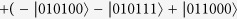 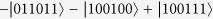 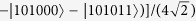	1	1	−1	−1	1	1	1	1	−1	−1	−1	1	1	1	−1

**Table 5:** The relation of the QPC protocol^18^ to our QSS protocol.

	QPC	QSS
Aim	Judge *M*_1_ = *M*_2_ = *M*_3_ or not	Share and recover *S*
Participants	*P*_1_ *P*_2_ and *P*_3_	*Boss* (Charlie), *Agent*_1_ (Alice), and *Agent*_2_ (Bob)
Quantum States	The BPB state and auxiliary decoy states	A specific state of the BPB-class/BPB-like-class state and auxiliary decoy states
Quantum Operations	(1) *P*_1_’*s preparation of* |Ψ_6*qb*_〉 *state*;	(1) *Charlie’s preparation of* 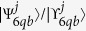 *state*;
	(2) *P*_1_’*s preparation of decoy states*;	(2) *Charlie’s preparation of decoy states*;
	(3) *P*_1_’*s transportation of* 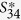 *and* 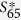 ;	(3) *Charlie’s transportation of S*_*A*2_ *and S*_*B*2_;
	(4) *P*_2_ *and P*_3_*'s measurement of decoy states*;	(4) *Alice and Bob’s measurement of decoy states*;
	(5) *P*_1_’*s measurement of sample* |Ψ_6*qb*_〉 *states*;	(5) *Three participants’ Bell basis measurement of* 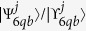 *state*.
	(6) *P*_2_ *and P*_3_’*s measurement of sample* |Ψ_6*qb*_〉 *states*;	
	(7) *Three participants’ Bell basis measurement of* |Ψ_6*qb*_〉 *state*.	
Quantum Equipment	quantum memory and quantum measurement device for each participant	quantum memory and quantum measurement device for each participant
Classical Operations	(1) *P*_1_’*s announcement of the exact position and corresponding measurement basis of decoy state*;	(1) *Charlie’s announcement of the exact position and corresponding measurement basis of decoy state*;
	(2) *P*_2_ *and P*_3_’*s announcement of measurement result about decoy state*;	(2) *Alice and Bob’s announcement of measurement result about decoy state*;
	(3) *P*_1_’*s analysis of eavesdropper’s existence*;	(3) *Charlie’s analysis of eavesdropper’s existence*;
	(4) *P*_2_ *and P*_3_’*s choose some sample states in all the* |Ψ_6*qb*_〉 *state*;	(4) *Charlie’s computation of CS*;
	(5) *P*_2_ *and P*_3_’*s announcement of the positions about the sample states*;	
	(6) *P*_2_ *and P*_3_’*s analysis of the authenticity of* |Ψ_6*qb*_〉 *state*;	
	(7) *P*_1_*/P*_2_*/P*_3_’*s computation of C*_1_*/C*_2_*/C*_3_;	
	(8) *P*_2_ *and P*_3_’*s transportation of C*_2_*,C*_3_;	(5) *Charlie’s transportation of CS*;
	(9) *P*_1_’*s judgement of whether M*_2_ = *M*_3_ *or not*;	(6) *Alice’s computation of S′*.
	(10) *P*_1_’*s announcement of judgement or P*_1_’*s computation of C*_13_;	
	(11) *P*_1_’*s transportation of C*_13_ *and C*_12_;	
	(12) *P*_2_’s (*P*_3_’s) *judgement of whether M*_1_ = *M*_3_ (M1 = M2) *or not*.	
Classical Equipment	Classical memory and calculator for each participant	Classical memory and calculator for each participant

**Table 6:** The correspondence of symbols in Table 5.

	QPC	QSS
3*Participants	*P*_1_	Charlie
	*P*_2_	Alice
	*P*_3_	Bob
2*States sequences	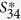	*S*_A2_
	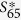	*S*_B2_

**Table 7:** The coupling between different modules in our QSS protocol.

	*Aim*	*P*	*QS*	*QO*	*QE*	*CO*	*CE*
*Aim*	N/A	+3	+1	+3	0	+3	0
*P*	−3	N/A	+1	+3	0	+3	0
*QS*	−1	−1	N/A	+1	0	+1	0
*QO*	−3	−3	−1	N/A	+2	+2	0
*QE*	0	0	0	−2	N/A	0	0
*CO*	−3	−3	−1	−2	0	N/A	+2
*CE*	0	0	0	0	0	−2	N/A
