# Supplementary material for: A kind of universal quantum secret sharing protocol
Source: Sci Rep. 2017 Jan 12;7:39845. doi: 10.1038/srep39845 (PMC5228055; doi:10.1038/srep39845)
Supplement: Supplementary Information [file srep39845-s1.pdf]

Supplementary information of *A kind of universal quantum secret sharing protocol*

Xiu-Bo Chen[1,3,*], Zhao Dou[1], Gang Xu[1,2,3], Xiao-Yu He[4], and Yi-Xian Yang[1]

[1]Information Security Center, State Key Laboratory of Networking and Switching Technology, Beijing University of Posts and Telecommunications, Beijing 100876, China
[2]School of Software Engineering, Beijing University of Posts and Telecommunications, Beijing 100876, China
[3]Department of Computer Science, University of Calgary, Calgary, Alberta, T2N 1N4, Canada
[4]School of Computer Science, Beijing University of Posts and Telecommunications, Beijing 100876, China
[*]corresponding Author: Xiu-Bo Chen, Email: flyover100@163.com

ABSTRACT

Entanglement of the BPB-class states are computed below. We firstly learn from the bipartite situation, and partially calculate the entanglements of aforementioned five states in Table 4 (of the main text). We call this kind of entanglement as pseudo entanglement. Then, we utilize Wei's tool[1] (Geometric measure) to calculate entanglement of the BPB-class states.

Supplementary information

The pseudo entanglement

Firstly, each particle of the states in Table 4 (of the main text) is analyzed. For the state $|\Psi^1_{6qb}\rangle$, we have:

$$\begin{aligned}
&\rho^1_1 = tr_{23456}(|\Psi^1_{6qb}\rangle \otimes \langle\Psi^1_{6qb}|)\\
&= \tfrac{1}{8}(|0\rangle \otimes \langle 0|tr(|00000\rangle \otimes \langle 00000| + |00101\rangle \otimes \langle 00101| + |10001\rangle \otimes \langle 10001| + |10100\rangle \otimes \langle 10100|)\\
&+|1\rangle \otimes \langle 1|tr(|01011\rangle \otimes \langle 01011| + |01110\rangle \otimes \langle 01110| + |11010\rangle \otimes \langle 11010| + |11111\rangle \otimes \langle 11111|))\\
&= \tfrac{1}{2}(|0\rangle \otimes \langle 0| + |1\rangle \otimes \langle 1|) = I_2/2.\\
&\rho^1_2 = \rho^1_3 = \rho^1_4 = \rho^1_5 = \rho^1_6 = \rho^1_1 = I_2/2.\\
&S(\rho^1_2) = S(\rho^1_3) = S(\rho^1_4) = S(\rho^1_5) = S(\rho^1_6) = S(\rho^1_1) = -tr(\rho^1_1 log_2 \rho^1_1) = 1.\\
&E_{p1}(|\Psi^1_{6qb}\rangle) = 1.
\end{aligned} \tag{1}$$

Here, $E_{p1}(*)$ means the pseudo entanglement of the state if only calculate one particle at one time.

Likewise,

$$E_{p1}(|\Psi^2_{6qb}\rangle) = E_{p1}(|\Psi^3_{6qb}\rangle) = E_{p1}(|\Psi^4_{6qb}\rangle) = E_{p1}(|\Psi^5_{6qb}\rangle) = E_{p1}(|\Psi^1_{6qb}\rangle) = 1. \tag{2}$$

Further, we group the six particles as pairs: (*1-st*, *2-nd*), (*3-rd*, *4-th*), (*5-th*, *6-th*). The pseudo entanglements of each pair are calculated.

$$\begin{aligned}
&\rho^j_{12} = tr_{3456}(|\Psi^j_{6qb}\rangle \otimes \langle\Psi^j_{6qb}|)\\
&= \tfrac{1}{16}(|\Phi^+\rangle \otimes \langle\Phi^+|tr(|\Phi^+\Phi^+\rangle\langle\Phi^+\Phi^+| + |\Phi^-\Phi^-\rangle\langle\Phi^-\Phi^-| + |\Psi^+\Psi^+\rangle\langle\Psi^+\Psi^+| + |\Psi^-\Psi^-\rangle\langle\Psi^-\Psi^-|)\\
&+|\Phi^-\rangle \otimes \langle\Phi^-|tr(|\Phi^+\Phi^-\rangle\langle\Phi^+\Phi^-| + |\Phi^-\Phi^+\rangle\langle\Phi^-\Phi^+| + |\Psi^+\Psi^-\rangle\langle\Psi^+\Psi^-| + |\Psi^-\Psi^+\rangle\langle\Psi^-\Psi^+|)\\
&+|\Psi^+\rangle \otimes \langle\Psi^+|tr(|\Phi^+\Psi^+\rangle\langle\Phi^+\Psi^+| + |\Phi^-\Psi^-\rangle\langle\Phi^-\Psi^-| + |\Psi^+\Phi^+\rangle\langle\Psi^+\Phi^+| + |\Psi^-\Phi^-\rangle\langle\Psi^-\Phi^-|)\\
&+|\Psi^-\rangle \otimes \langle\Psi^-|tr(|\Phi^+\Psi^-\rangle\langle\Phi^+\Psi^-| + |\Phi^-\Psi^+\rangle\langle\Phi^-\Psi^+| + |\Psi^+\Phi^-\rangle\langle\Psi^+\Phi^-| + |\Psi^-\Phi^+\rangle\langle\Psi^-\Phi^+|))\\
&= \tfrac{1}{4}(|\Phi^+\rangle \otimes \langle\Phi^+| + |\Phi^-\rangle \otimes \langle\Phi^-| + |\Psi^+\rangle \otimes \langle\Psi^+| + |\Psi^-\rangle \otimes \langle\Psi^-|)\\
&= I_4/4.
\end{aligned} \tag{3}$$

$$\begin{aligned}
&\rho^j_{34} = \rho^j_{56} = \rho^j_{12} = I_4/4.\\
&S(\rho^j_{34}) = S(\rho^j_{56}) = S(\rho^j_{12}) = -tr(\rho^j_{12} log_2 \rho^j_{12}) = 1.\\
&E_{p2}(|\Psi^j_{6qb}\rangle) = 1.
\end{aligned} \tag{4}$$

Here, $E_{p2}(*)$ means the pseudo entanglement of the state if calculate a pair of particles at one time. That is to say, for every state in the class, $E_{p2}(|\Psi^j_{6qb}\rangle)=1$. This is another common character of these states. From this point of view, the entanglements of states are high.

By the way, though not all of the states in the class are maximally entangled, the reduced density matrix of Alice (Bob, Charlie)'s particles is $I_4/4$ at all times. According to analysis in Section *Security Analysis*, she (he) also cannot deduce any valuable information. Our protocol is safe.

Geometric measure of the BPB-class states

In Eq. (7) (of the main text), the states are written in Bell basis. For the sake of the convenient calculation, the states are needed to be rewritten in Z basis. Since coefficients of the states are complex, we need to divide the items in Eq. (7) (of the main text) into groups (in Table S1). The items, whose measurement results in Z basis produce the same statistics, are in the same group.

Table S1. The groups of items

1-st group	*2-nd* group	*3-rd* group	*4-th* group
$\lvert\Phi^+\Phi^+\Phi^+\rangle + a_1\lvert\Phi^+\Phi^-\Phi^-\rangle$ $+a_4\lvert\Phi^-\Phi^+\Phi^-\rangle + a_5\lvert\Phi^-\Phi^-\Phi^+\rangle$	$a_2\lvert\Phi^+\Psi^+\Psi^+\rangle + a_3\lvert\Phi^+\Psi^-\Psi^-\rangle$ $+a_6\lvert\Phi^-\Psi^+\Psi^-\rangle + a_7\lvert\Phi^-\Psi^-\Psi^+\rangle$	$a_8\lvert\Psi^+\Phi^+\Psi^+\rangle + a_9\lvert\Psi^+\Phi^-\Psi^-\rangle$ $+a_{12}\lvert\Psi^-\Phi^+\Psi^-\rangle + a_{13}\lvert\Psi^-\Phi^-\Psi^+\rangle$	$a_{10}\lvert\Psi^+\Psi^+\Phi^+\rangle + a_{11}\lvert\Psi^+\Psi^-\Phi^-\rangle$ $+a_{14}\lvert\Psi^-\Psi^+\Phi^-\rangle + a_{15}\lvert\Psi^-\Psi^-\Phi^+\rangle$

We rewrite the state in the 1-st group individually.

$$\begin{aligned}
|\Phi^+\Phi^+\Phi^+\rangle &= \tfrac{1}{2\sqrt{2}}(|000000\rangle+|111111\rangle+|000011\rangle+|111100\rangle+|001100\rangle+|110011\rangle+|001111\rangle+|110000\rangle),\\
|\Phi^+\Phi^-\Phi^-\rangle &= \tfrac{1}{2\sqrt{2}}(|000000\rangle+|111111\rangle-|000011\rangle-|111100\rangle-|001100\rangle-|110011\rangle+|001111\rangle+|110000\rangle),\\
|\Phi^-\Phi^+\Phi^-\rangle &= \tfrac{1}{2\sqrt{2}}(|000000\rangle+|111111\rangle-|000011\rangle-|111100\rangle+|001100\rangle+|110011\rangle-|001111\rangle-|110000\rangle),\\
|\Phi^-\Phi^-\Phi^+\rangle &= \tfrac{1}{2\sqrt{2}}(|000000\rangle+|111111\rangle+|000011\rangle+|111100\rangle-|001100\rangle-|110011\rangle-|001111\rangle-|110000\rangle).
\end{aligned} \tag{5}$$

Then, superposition of these four state is

$$\begin{aligned}
&a_0|\Phi^+\Phi^+\Phi^+\rangle+a_1|\Phi^+\Phi^-\Phi^-\rangle+a_4|\Phi^-\Phi^+\Phi^-\rangle+a_5|\Phi^-\Phi^-\Phi^+\rangle\\
&=\tfrac{1}{C_a}[(a_0+a_1+a_4+a_5)|000000\rangle+(a_0+a_1+a_4+a_5)|111111\rangle\\
&+(a_0-a_1-a_4+a_5)|000011\rangle+(a_0-a_1-a_4+a_5)|111100\rangle\\
&+(a_0-a_1+a_4-a_5)|001100\rangle+(a_0-a_1+a_4-a_5)|110011\rangle\\
&+(a_0+a_1-a_4-a_5)|001111\rangle+(a_0+a_1-a_4-a_5)|110000\rangle].
\end{aligned} \tag{6}$$

Here, C_a represents normalization coefficient. Let $i_1i_2i_3i_4i_5i_6\in\{000000,000011,001100,001111\}$, $\bar{i_j}=1\oplus i_j$ for $1\le j\le 6$. The coefficient of $|i_1i_2i_3i_4i_5i_6\rangle$ and $|\bar{i_1}\bar{i_2}\bar{i_3}\bar{i_4}\bar{i_5}\bar{i_6}\rangle$ are the same.

Likewise, in the other groups, similar results can be found. Overall, the coefficient of $|i_1i_2i_3i_4i_5i_6\rangle$ and $|\bar{i_1}\bar{i_2}\bar{i_3}\bar{i_4}\bar{i_5}\bar{i_6}\rangle$ are the same for $i_1i_2i_3i_4i_5i_6\in\{000000,000011,001100,001111,000101,000110,001001,001010,010001,010010,011101,011110,010100,010111,011000,011011\}$.

So, the states $|\Psi^j_{6qb}\rangle$ can be re-written in Z basis as:

$$\begin{aligned}
|\Psi^j_{6qb}\rangle = [&(e_1|000000\rangle+e_2|000011\rangle+e_3|001100\rangle+e_4|001111\rangle+e_4|110000\rangle+e_3|110011\rangle+e_2|111100\rangle+e_1|111111\rangle)\\
&(+b_1|000101\rangle+b_2|000110\rangle+b_3|001001\rangle+b_4|001010\rangle+b_4|110101\rangle+b_3|110110\rangle+b_2|111001\rangle+b_1|111010\rangle)\\
&(+c_1|010001\rangle+c_2|010010\rangle+c_3|011101\rangle+c_4|011110\rangle+c_4|100001\rangle+c_3|100010\rangle+c_2|101101\rangle+c_1|101110\rangle)\\
&(+d_1|010100\rangle+d_2|010111\rangle+d_3|011000\rangle+d_4|011011\rangle+d_4|100100\rangle+d_3|100111\rangle+d_2|101000\rangle+d_1|101011\rangle)]_{123456}.
\end{aligned} \tag{7}$$

Here, $e_1=1/(2C_a)*(a_0+a_1+a_4+a_5)$, $e_2=1/(2C_a)*(a_0-a_1-a_4+a_5)$, $e_3=1/(2C_a)*(a_0-a_1+a_4-a_5)$, $e_4=1/(2C_a)*(a_0+a_1-a_4-a_5)$. And b_i, c_i, $d_i (i=1,2,3,4)$ represent the other coefficients. Since $a_i\in\{1,-1\}$, and different a_i are independent, it's easy to verify that:

$$\begin{aligned}
&\{b_1,b_2,b_3,b_4\},\{c_1,c_2,c_3,c_4\},\{d_1,d_2,d_3,d_4\}\in\\
&\{\{1/(2\sqrt{2}),0,0,0\},\{-1/(2\sqrt{2}),0,0,0\},\{1/(4\sqrt{2}),1/(4\sqrt{2}),1/(4\sqrt{2}),-1/(4\sqrt{2})\},\\
&\{1/(4\sqrt{2}),-1/(4\sqrt{2}),-1/(4\sqrt{2}),-1/(4\sqrt{2})\}\}.
\end{aligned} \tag{8}$$

Since $a_0 = 1$, we also have

$$\{e_1, e_2, e_3, e_4\} \in \{\{1/(2\sqrt{2}), 0, 0, 0\}, \{1/(4\sqrt{2}), 1/(4\sqrt{2}), 1/(4\sqrt{2}), -1/(4\sqrt{2})\}\}. \tag{9}$$

When $\{e_1, e_2, e_3, e_4\}$ has a certain value, for example, $\{1/(2\sqrt{2}), 0, 0, 0\}$, we mean that two sets are the same, instead of the value of corresponding elements.

Now, suppose that the state $|\phi\rangle = (\cos\theta_i|0\rangle + \sin\theta_i|1\rangle)^{\otimes 6}$ ($\theta_i \in (0, \pi]$). Then, we calculate the inner product.

$$\begin{aligned}
&|\psi\rangle = |\Psi^j_{6qb}\rangle \\
&|\phi\rangle = (\cos\theta_i|0\rangle + \sin\theta_i|1\rangle)^{\otimes 6} \\
&\langle\phi|\psi\rangle = e_1 \cos\theta_1 \cos\theta_2 \cos\theta_3 \cos\theta_4 \cos\theta_5 \cos\theta_6 + b_1 \cos\theta_1 \cos\theta_2 \cos\theta_3 \cos\theta_5 \sin\theta_4 \sin\theta_6 \\
&+b_2 \cos\theta_1 \cos\theta_2 \cos\theta_3 \cos\theta_6 \sin\theta_4 \sin\theta_5 + b_3 \cos\theta_1 \cos\theta_2 \cos\theta_4 \cos\theta_5 \sin\theta_3 \sin\theta_6 \\
&+b_4 \cos\theta_1 \cos\theta_2 \cos\theta_4 \cos\theta_6 \sin\theta_3 \sin\theta_5 + c_1 \cos\theta_1 \cos\theta_3 \cos\theta_4 \cos\theta_5 \sin\theta_2 \sin\theta_6 \\
&+c_2 \cos\theta_1 \cos\theta_3 \cos\theta_4 \cos\theta_6 \sin\theta_2 \sin\theta_5 + c_3 \cos\theta_2 \cos\theta_3 \cos\theta_4 \cos\theta_6 \sin\theta_1 \sin\theta_5 \\
&+c_4 \cos\theta_2 \cos\theta_3 \cos\theta_4 \cos\theta_5 \sin\theta_1 \sin\theta_6 + d_1 \cos\theta_1 \cos\theta_3 \cos\theta_5 \cos\theta_6 \sin\theta_2 \sin\theta_4 \\
&+d_2 \cos\theta_2 \cos\theta_4 \cos\theta_5 \cos\theta_6 \sin\theta_1 \sin\theta_3 + d_3 \cos\theta_1 \cos\theta_4 \cos\theta_5 \cos\theta_6 \sin\theta_2 \sin\theta_3 \\
&+d_4 \cos\theta_2 \cos\theta_3 \cos\theta_5 \cos\theta_6 \sin\theta_1 \sin\theta_4 + e_2 \cos\theta_1 \cos\theta_2 \cos\theta_3 \cos\theta_4 \sin\theta_5 \sin\theta_6 \\
&+e_3 \cos\theta_1 \cos\theta_2 \cos\theta_5 \cos\theta_6 \sin\theta_3 \sin\theta_4 + e_4 \cos\theta_3 \cos\theta_4 \cos\theta_5 \cos\theta_6 \sin\theta_1 \sin\theta_2 \\
&+b_1 \cos\theta_4 \cos\theta_6 \sin\theta_1 \sin\theta_2 \sin\theta_3 \sin\theta_5 + b_2 \cos\theta_4 \cos\theta_5 \sin\theta_1 \sin\theta_2 \sin\theta_3 \sin\theta_6 \\
&+b_3 \cos\theta_3 \cos\theta_6 \sin\theta_1 \sin\theta_2 \sin\theta_4 \sin\theta_5 + b_4 \cos\theta_3 \cos\theta_5 \sin\theta_1 \sin\theta_2 \sin\theta_4 \sin\theta_6 \\
&+c_1 \cos\theta_2 \cos\theta_6 \sin\theta_1 \sin\theta_3 \sin\theta_4 \sin\theta_5 + c_2 \cos\theta_2 \cos\theta_5 \sin\theta_1 \sin\theta_3 \sin\theta_4 \sin\theta_6 \\
&+c_3 \cos\theta_1 \cos\theta_5 \sin\theta_2 \sin\theta_3 \sin\theta_4 \sin\theta_6 + c_4 \cos\theta_1 \cos\theta_6 \sin\theta_2 \sin\theta_3 \sin\theta_4 \sin\theta_5 \\
&+d_1 \cos\theta_2 \cos\theta_4 \sin\theta_1 \sin\theta_3 \sin\theta_5 \sin\theta_6 + d_2 \cos\theta_1 \cos\theta_3 \sin\theta_2 \sin\theta_4 \sin\theta_5 \sin\theta_6 \\
&+d_3 \cos\theta_2 \cos\theta_3 \sin\theta_1 \sin\theta_4 \sin\theta_5 \sin\theta_6 + d_4 \cos\theta_1 \cos\theta_4 \sin\theta_2 \sin\theta_3 \sin\theta_5 \sin\theta_6 \\
&+e_2 \cos\theta_5 \cos\theta_6 \sin\theta_1 \sin\theta_2 \sin\theta_3 \sin\theta_4 + e_3 \cos\theta_3 \cos\theta_4 \sin\theta_1 \sin\theta_2 \sin\theta_5 \sin\theta_6 \\
&+e_4 \cos\theta_1 \cos\theta_2 \sin\theta_3 \sin\theta_4 \sin\theta_5 \sin\theta_6 + e_1 \sin\theta_1 \sin\theta_2 \sin\theta_3 \sin\theta_4 \sin\theta_5 \sin\theta_6.
\end{aligned} \tag{10}$$

Here, the built-in function *FindMaxmum* of the mathematics software Mathematica (version 10.1) is utilized to calculate all the 2^{15} values of $\Lambda_{\max} = \max_\phi \parallel \langle\phi|\psi\rangle \parallel$. Since it's difficult to compute the maximum of multivariable function, and the results depend on the initial guesses of searching, *FindMaxmum* is performed for 8 rounds with different initial guesses. Then, we get the maximum among these 8 maximum values. It means that our results are very close to the real $\Lambda_{\max}$. Here, The eight groups of initial guesses are shown in Table S2.

Table S2. The initial guesses of function *FindMaxmum*

	θ_1	θ_2	θ_3	θ_4	θ_5	θ_6
Searching range	$(0,\pi]$	$(0,\pi]$	$(0,\pi]$	$(0,\pi]$	$(0,\pi]$	$(0,\pi]$
round-1	$\pi/2$	1.6	1.5	1.4	1.7	1.8
round-2	$\pi/2$	1	2	1.5	1.6	1.7
round-3	0.5	1.1	1.6	1.7	2.5	2
round-4	0.8	1.6	0.9	2.6	2	1.7
round-5	2.4	1	2.2	1.2	1.6	1.7
round-6	0.4	2.9	2.8	0.3	2	1.6
round-7	1.7	0.4	2.9	2.8	1.5	0.3
round-8	0.2	2.6	2.8	0.4	3	0.5

The distribution of all the 2^{15} $\Lambda_{\max}$ is given in Figure S1.

Simple statistics tells us, the average of these values is 0.391, the minimum is 0.25, and the mode is 0.354. There are 6949 states, whose value of $\Lambda_{\max}$ is 0.354. Besides that, from this figure, we also can see that the values between 0.385∼0.39 are also highly significant. There are more than 1/3 states in this interval.

Further, all the E_{sin^2} are calculated, and the distribution is also shown in Figure S2.

Similarly, the maximum of E_{sin^2} is 0.9375. In fact, there are 128 states whose value equals it. These highly entangled states should be concerned more. An example of them is shown in Eq. (11).

$$\begin{aligned}
|\Psi^6_{6qb}\rangle = \; & \tfrac{1}{\sqrt{32}}[(|000000\rangle + |000011\rangle + |001100\rangle - |001111\rangle - |110000\rangle + |110011\rangle + |111100\rangle + |111111\rangle) \\
& +(|000101\rangle + |000110\rangle + |001001\rangle - |001010\rangle - |110101\rangle + |110110\rangle + |111001\rangle + |111010\rangle) \\
& +(|010001\rangle + |010010\rangle - |011101\rangle + |011110\rangle + |100001\rangle - |100010\rangle + |101101\rangle + |101110\rangle) \\
& +(-|010100\rangle - |010111\rangle + |011000\rangle - |011011\rangle - |100100\rangle + |100111\rangle - |101000\rangle - |101011\rangle)]_{123456}.
\end{aligned} \tag{11}$$

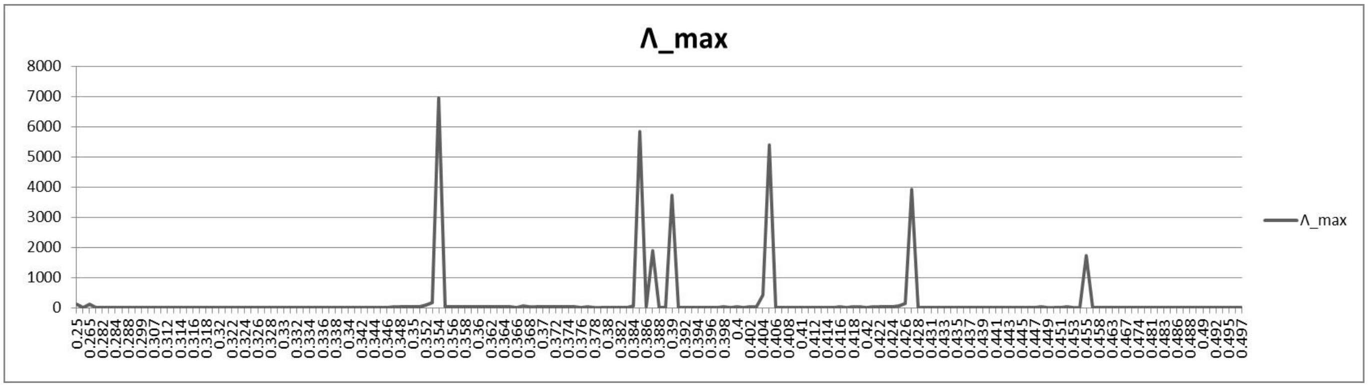

Figure S1. The distribution of Λ_{max}

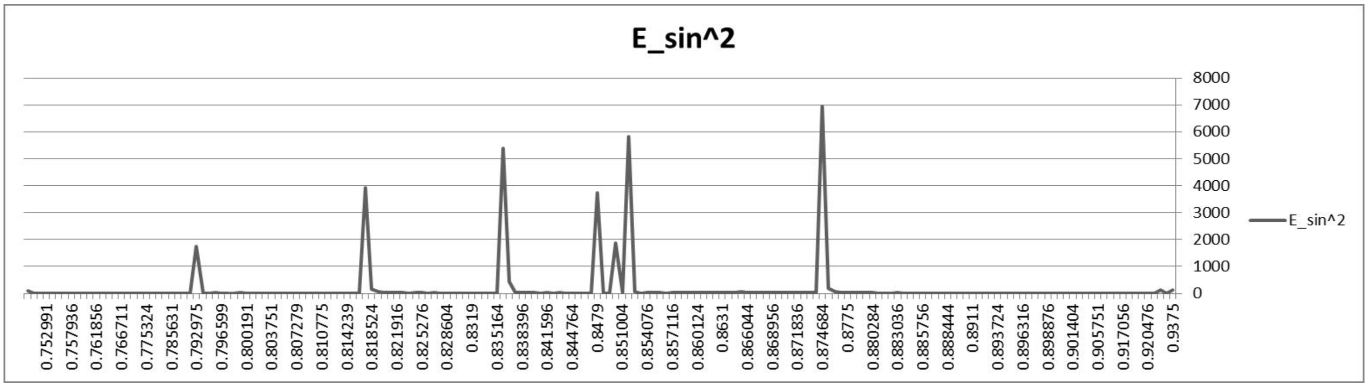

Figure S2. The distribution of E_{sin^2}

In conclusion, above calculations will help us to know more about the BPB-class states, and select highly entangled state for quantum information processing.

References

1. Wei T.C., Goldbart P.M. Geometric measure of entanglement and applications to bipartite and multipartite quantum states. *Physical Review A*, **68**, 042307 (2003).
